# Factors Affecting Cardiovascular Risk in Children, Adolescents, and Young Adults with Type 1 Diabetes

**DOI:** 10.1155/2019/9134280

**Published:** 2019-05-16

**Authors:** Ingrida Stankute, Rimante Dobrovolskiene, Evalda Danyte, Dovile Razanskaite-Virbickiene, Edita Jasinskiene, Giedre Mockeviciene, Dalia Marciulionyte, Valerie M. Schwitzgebel, Rasa Verkauskiene

**Affiliations:** ^1^Medical Academy, Lithuanian University of Health Sciences, Kaunas, Lithuania; ^2^Institute of Endocrinology, Lithuanian University of Health Sciences, Kaunas, Lithuania; ^3^Pediatric Endocrine and Diabetes Unit, Department of Pediatrics, Gynecology and Obstetrics, University Hospitals of Geneva, 1211 Geneva, Switzerland; ^4^Diabetes Center of the Faculty of Medicine, University of Geneva, 1211 Geneva, Switzerland

## Abstract

Cardiovascular risk and obesity are becoming major health issues among individuals with type 1 diabetes (T1D). The aim of this study was to evaluate cardiovascular risk factors and obesity in youth with T1D in Lithuania. *Methods*. 883 patients under 25 years of age with T1D for at least 6 months were investigated. Anthropometric parameters, blood pressure, and microvascular complications were evaluated, and the lipid profile and HbA1c were determined for all patients. *Results*. Study subjects' mean HbA1c was 8.5 ± 2%; 19.5% were overweight and 3.6% obese. Hypertension and dyslipidemia were diagnosed in 29.8% and 62.6% of participants, respectively. HbA1c concentration was directly related to levels of total cholesterol (*r* = 0.274, *p* < 0.001), LDL (*r* = 0.271, *p* < 0.001), and triglycerides (*r* = 0.407, *p* < 0.001) and inversely associated with levels of HDL (*r* = 0.117, *p* = 0.001). Prevalence of dyslipidemia increased with duration of diabetes (*p* < 0.05). Hypertension was more prevalent in overweight and obese compared to normal-weight patients (40.6 and 65.6 vs. 25.6%, respectively, *p* < 0.001). Frequency of microvascular complications was higher among patients with dyslipidemia (27.2 vs. 18.8%, *p* = 0.005) and among those with hypertension (25.9 vs. 23.2%, *p* < 0.001). *Conclusion*. The frequency of cardiovascular risk factors is high in youth with T1D and associated with diabetes duration, obesity, and metabolic control.

## 1. Introduction

The main cause of death in European countries is cardiovascular diseases (CVD) [1]. Several studies showed that atherosclerosis presents more frequently in people with diabetes. This is usually explained by persistently elevated glucose levels in the blood [2, 3].

CVD tend to present at a younger age in patients with diabetes than in the general population [4]. The SEARCH for Diabetes in Youth Study showed that significant complications severely affect the quality of life of diabetics early in their life [5]. Therefore, adolescence and young adulthood are the best times for actions to lower cardiovascular risk [6].

The other growing issue in type 1 diabetes (T1D) patients is obesity, which aggravates the risk of hypertension and dyslipidemia [7].

The aim of our study was to analyze the risk factors for CVD in children and young adults under the age of 25 years with T1D in Lithuania.

## 2. Materials and Methods

### 2.1. Subjects

The presented cohort included 883 subjects from the recent joint Lithuanian-Swiss project “Genetic Diabetes in Lithuania,” which had 1209 subjects overall, covering all children and 70% of young adult (under the age 25) patients with T1D in Lithuania, described previously [8].

This analysis included patients with established diabetes longer than 6 months and treated with insulin: 590 of them (66.8%) were from 1- to 17-year-old children and adolescents, and 293 are young adults (33.2%) from 18 to 25 years old. Subjects with diabetes duration less than 6 months were not included in this analysis due to the weight fluctuation and metabolic instability that are usually seen at the onset and initial therapy of diabetes [9]. None of the study participants were taking any medication affecting body composition, blood pressure, or renal function.

At the time of involvement in the project, data about age, duration of T1D, insulin delivery method (pump/injection), and total daily insulin dose (U/kg/d) were collected and clinical and laboratory assessments done. The participants were consulted by a single ophthalmologist for evaluation of diabetic retinopathy and a pediatric or adult neurologist for assessment of diabetic neuropathy.

The Lithuanian Bioethics Committee granted the approval for this biomedical research (No. BE-2-5). Subject information forms and informed consent forms were signed by each participant or official representative.

### 2.2. Clinical Assessment and Examination

Anthropometric parameters were measured by clinical nurses at the Endocrinology Department of the Hospital of Lithuanian University of Health Sciences. At this department, the Harpenden Stadiometer (Holtain, Crymych, UK) is used for height measurement. Patients' height was measured to the nearest ±0.1 cm three times, then the average was estimated for analyses. seca 700 medical scales (seca GmbH & Co. KG) were used for weight in kg, with precision of 0.1 kg.

For body mass index (BMI), the equation weight in kg/recumbent length or standing height in m^2^ was used.

For participants under 19 years, a BMI *z*-score was evaluated according to age and gender using the references of the World Health Organization (WHO); for participants aged 19 to 25 years, a BMI *z*-score was evaluated according to the references of WHO for 19-year-old individuals, assuming that their linear growth is over.

For all participants, normal weight was defined as BMI ranging from -2 standard deviations (SD) to less or equal to +1 SD (which corresponds to BMI 25 kg/m^2^ at 19 years); overweight was defined as BMI less than +2 SD (which corresponds to BMI 30 kg/m^2^ at 19 years), obese as BMI>+2 SD, and underweight as BMI < ‐2 SD.

A medical measuring tape was used for waist and hip measurements. The approximate middle point between the lower margin of the last palpable rib and the top of the iliac crest was measured, to the nearest ±0.1 cm, for waist circumference [10]. Hip circumference was measured at the widest point of the buttocks, to the nearest ±0.1 cm.

In analyses, the waist-hip ratio (waist circumference (cm)/hip circumference (cm)) and waist-to-height ratio (WtHR) (waist circumference (cm)/height (cm)) were used. The waist‐hip ratio ≥ 0.90 cm for men and ≥0.85 cm for women were considered significantly increased [10]. WtHR < 0.5 cm was considered as optimal [11].

Arterial blood pressure for children and adults was measured after sitting in silence for 5 min using an oscillometric sphygmomanometer in the left arm with appropriate cuff size. For children, “The Fourth Report from the National High Blood Pressure Education Program (NHBPEP) Working Group on Children and Adolescents” guidelines was used to classify measurements of arterial blood pressure: “Normal BP was defined as systolic blood pressure (SBP) and diastolic blood pressure (DBP) less than the 90th percentile for sex, age, and height. Hypertension was defined as average SBP or DBP that was greater than or equal to the 95th percentile for sex, age, and height on at least three separate occasions” [12].

Measurements of arterial blood pressure in adults were classified according to the American Heart Association (AHA) guidelines: “Normal blood pressure defined as SBP <120 mmHg and DBP <80 mmHg, hypertension beginning at 140/90 mmHg and higher” [13].

### 2.3. Laboratory Analyses

Glycosylated hemoglobin (HbA1c) and lipid profiles were measured by the UniCel DxC 800 Synchron system (Beckman Coulter, USA). The normal cutoff values of HbA1c were 4-6% (20 mmol/mol-42 mmol/mol). International Society for Pediatric and Adolescent Diabetes (ISPAD) guidelines were used to define optimal metabolic control when HbA1c < 7.5% (58 mmol/mol) for children and adolescents and <7% for young adults with reference to American Diabetes Association guidelines [14, 15].

Normal values for low-density lipoprotein cholesterol (LDL), high-density lipoprotein cholesterol (HDL), and triglycerides (Tg) were defined as <2.6 mmol/l, >1.1 mmol/l, and <1.7 mmol/l, respectively [14, 16]. Normal values for total cholesterol were defined as <5.2 mmol/l for patients ≥16 yrs and <5.5 mmol/l for children under 16 yrs. If at least one lipid value was abnormal, dyslipidemia was considered to be present.

### 2.4. Evaluation of Microvascular Diabetes Complications

All participants were screened for microvascular diabetes complications at the same Endocrinology Department. A single diabetes ophthalmologist and an adult/pediatric neurologist consulted with all diabetes patients for the presence of retinopathy and neuropathy, respectively. Diabetic retinopathy was identified from stereoscopic fundal examination. Sensations for vibration, pressure, and temperature were evaluated for each patient, and all of them were surveyed with the Michigan Neuropathy Screening Questionnaire. If ≥2 of these tests were abnormal, peripheral neuropathy was diagnosed [8, 17, 18].

For diabetic kidney damage, a 24-hour urine albumin excretion rate (AER) was evaluated. AER < 30 mg/24 h was defined as normal, 30-300 mg/24 h—microalbuminuria, and >300 mg/24 h—macroalbuminuria [19].

### 2.5. Statistical Analyses

The IBM SPSS Statistics Base version 22.0 was used for statistical analysis of the data. In case of normal data distribution, Student's 2-tailed *t* test, *χ*^2^ statistics, and parametric one-way ANOVA were used. For nonnormally distributed data, the Mann-Whitney *U* test was used, and for ordinal data, Kruskal-Wallis one-way ANOVA was used. For estimation of trends, linear regression models were used. For testing the hypothesis about relationships between dichotomous-dependent variables and continuous predictors, binary logistic regression analysis was carried out. *p* values <0.05 were considered as statistically significant. All *p* values were two-sided.

## 3. Results

### 3.1. General Characteristics and Weight Status

Of the 883 subjects enrolled for the current analysis, 49.2% (*n* = 434) were males. The mean age of study subjects was 16.2 ± 5.6 yrs. The average diabetes duration was 6.7 ± 4.8 yrs (0.5-24.73, median 5.6 yrs). The distribution of patients by duration of T1D was as follows: 0.5-4 years 45.4% (*n* = 401), 5-9 years 30.9% (*n* = 273), 10-14 years 17.9% (*n* = 158), 15-19 years 4.3% (*n* = 38), and ≥20 years 1.5% (*n* = 13).

**Figure 1:**
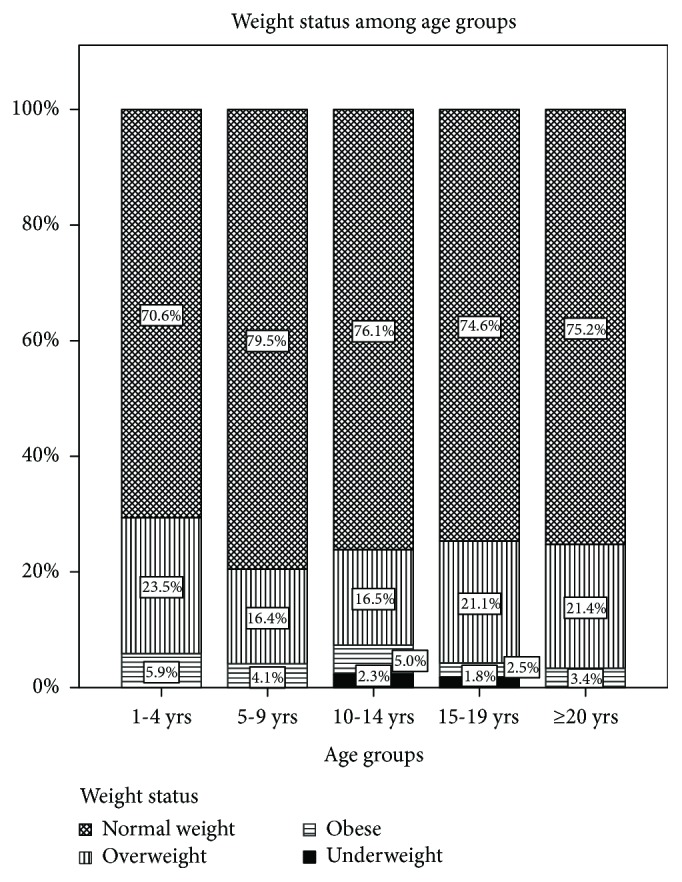
Normal weight, overweight, obese, and underweight frequency among different age groups.

The mean BMI *z*-score in the whole cohort was 0.29 ± 0.99. 75.8% of study subjects (*n* = 666) were of normal weight, 19.5% (*n* = 171) overweight, 3.6% (*n* = 32) obese, and 1.1% (*n* = 10) underweight. The distribution of weight status among different age groups is shown in Figure 1 (*p* > 0.05). 20.5% of females and 18.3% of males were overweight (*p* > 0.05), and 3.3% of females and 3.9% of males were obese (*p* > 0.05).

Clinical characteristics according to weight group are shown in Table 1. Hypertension was more frequent among overweight and obese patients than among normal weight patients (40.6% and 65.6% vs. 25.6%, respectively, *p* < 0.05).

21.8% (*n* = 192) of patients had WtHR higher than 0.5. As expected, overweight and obese individuals had higher WtHR than normal-weight subjects (0.49 ± 0.04 and 0.55 ± 0.06 vs. 0.44 ± 0.15, respectively, *p* < 0.001). However, no significant differences in the waist-hip ratio were found comparing different-weight-status participants.

### 3.2. Glycemic and Metabolic Control

Study subjects' mean HbA1c was 8.5 ± 2% (69.2 ± 2 mmol/mol). 32.7% (*n* = 289) of patients had optimal glycemic control. The best glycemic control was recorded in the group of youngest patients: patients aged 1-4 yrs and 5-9 yrs had significantly lower HbA1c compared to patients aged 10-14 yrs, 15-19 yrs, and ≥20 yrs (7.3 ± 1% and 7.5 ± 1.2% vs. 8.5 ± 1.9%, 8.9 ± 2.1%, and 8.6 ± 1.9%, respectively, *p* < 0.05). In all age groups, females had significantly higher HbA1c than males, except in the youngest 1-4 yrs of age group (Table 2).

The average insulin dose was 0.83 ± 0.3 U/kg/d for the whole cohort. Adjusted for diabetes duration, patients with optimal glycemic control had a lower insulin dose compared to patients with suboptimal HbA1c (0.76 ± 0.31 U/kg/d vs. 0.87 ± 0.29 U/kg/d, respectively, *p* < 0.001). In the whole cohort, 30.2% of patients were on insulin pumps. HbA1c of patients treated with insulin pumps and multiple daily injections (MDI) was 8.5 ± 2% and 8.4 ± 1.8%, respectively, *p* > 0.05; also, there was a similar proportion of subjects with optimal glycemic control in insulin pump users or those on MDI (29.4% and 30.6%, respectively, *p* > 0.05).

Dyslipidemia was found in 62.6% (*n* = 552) of the total cohort. Increased total cholesterol was found in 28.2% (*n* = 249) of patients, increased LDL in 54.9% (*n* = 484), increased Tg in 9.2% (*n* = 81), and decreased HDL in 13.5% (*n* = 119) of patients. The frequency of dyslipidemia was similar among all age groups: 50% (*n* = 8) among patients aged 1-4 years, 52% (*n* = 62)—5-9 years, 64.7% (*n* = 141)—10-14 years, 64.4% (*n* = 183)—15-19 years, and 64.7% (*n* = 156)—≥20 years.

Dyslipidemia was more frequent in the group with poor glycemic control compared to that with optimal glycemic control (66.9% vs. 53.6%, respectively, *p* < 0.001). Also, the HbA1c level was significantly higher in patients with dyslipidemia compared to patients with a normal lipid profile (8.8 ± 2.1% vs. 8 ± 1.7%, respectively, *p* < 0.001). A significantly higher prevalence of dyslipidemia was found in patients with WtHR exceeding 0.5 compared to subjects whose WtHR was less than 0.5 (73.4% vs. 26.6%, respectively, *p* < 0.001).

**Figure 2:**
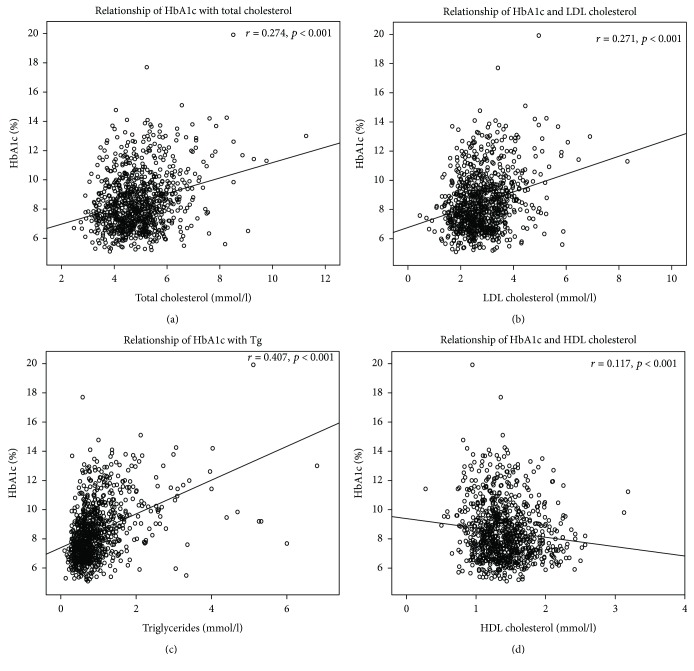
Correlations between levels of glycosylated hemoglobin (HbA1c) and lipids: (a) relationship of HbA1c and total cholesterol, (b) relationship of HbA1c and low density-lipoprotein cholesterol (LDL), (c) relationship of HbA1c and triglycerides (Tg), and (d) relationship of HbA1c and high density-lipoprotein cholesterol (HDL).

Significant direct correlations between the levels of HbA1c and total cholesterol, LDL, and Tg and reverse correlation between the levels of HbA1c and HDL were found (Figure 2).

A logistic regression was carried out to assess the predictors for the likelihood that the patient would or would not have dyslipidemia. Binary variable “Dyslipidemia” was coded as “1” if present and “0” if absent. The full model containing continuous predictors, HbA1c levels and WtHR, was statistically significant, *χ*^2^ = 39.473, *p* < 0.001, indicating that the model was able to differentiate between patients with and without dyslipidemia. Analysis of predictors on the probability of dyslipidemia is shown in Table 3.

The logistic regression model could be expressed as

(1) $$\begin{array}{c} \text{Probability}\left(\text{Dyslipidemia}\right)=\frac{1}{1+e^{-z}},\;z=-3.525+0.207*X_{1}+5.113*X_{2}, \end{array}$$

where *e* is the base of the natural logarithm, *X*_1_ is HbA1c, and *X*_2_ is WtHR.

Overall model predictions were successful in 62.1%. Binary logistic regression indicated that HbA1c and WtHR are significant predictors of likelihood of dyslipidemia. Cook's distances for the model ranged from a minimum of 0.0058 to a maximum of 0.0907. The maximum values of DFBETA for HbA1c and WtHR were 0.003 and 0.2, respectively.

**Figure 3:**
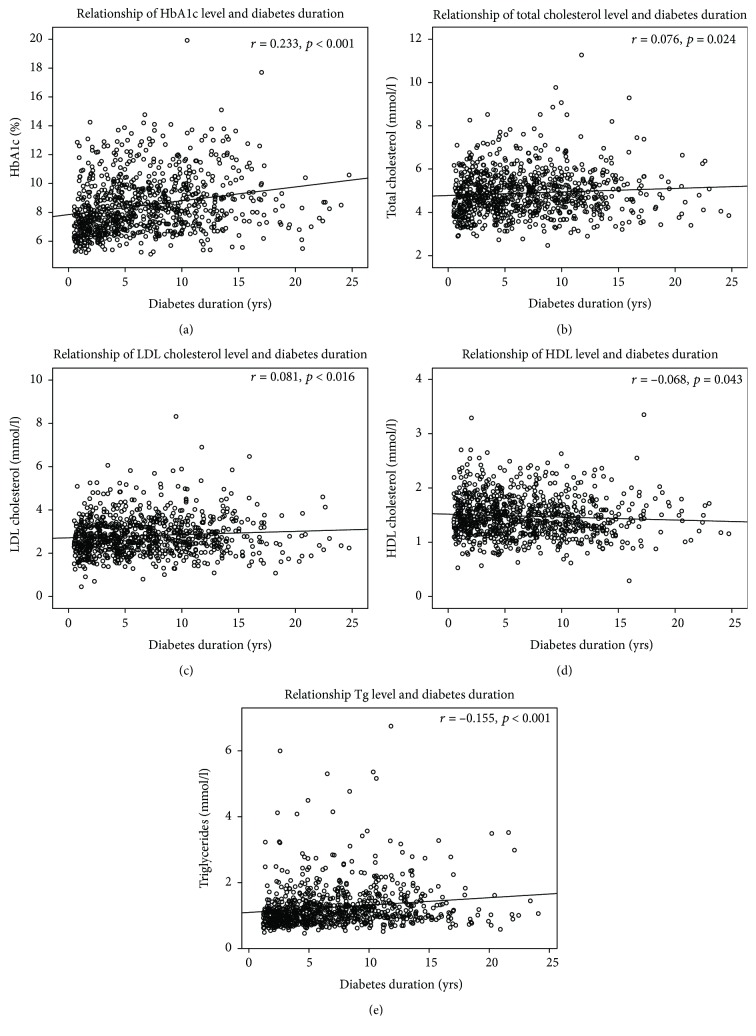
Correlations between diabetes and metabolic control parameters: (a) relationship of diabetes duration and glycosylated hemoglobin (HbA1c), (b) relationship of diabetes duration and total cholesterol, (c) relationship of diabetes duration and low density-lipoprotein cholesterol (LDL), (d) relationship of diabetes duration and high density-lipoprotein cholesterol (HDL), and (e) relationship of diabetes duration and triglycerides (Tg).

Impairment of all lipid fractions' metabolism and glycemic control was dependent on diabetes duration. The linear regression models are presented in Figure 3, showing levels of HbA1c, total cholesterol, LDL cholesterol, and Tg directly, and HDL cholesterol concentrations negatively related with duration of diabetes.

In linear regression models, controlled for diabetes duration and glycemic control, BMI *z*-scores were weakly but significantly negatively associated with HDL (*r* = −0.095, *p* = 0.005) and directly related to LDL and Tg concentrations (*r* = 0.086 and *p* = 0.011 and *r* = 0.088 and *p* = 0.009, respectively).

### 3.3. Blood Pressure and Microvascular Complications

Hypertension was diagnosed in 29.8% (*n* = 263) of patients. It was more frequent among males compared to females (34.5% vs. 26.1%, *p* = 0.007). Hypertension was diagnosed more often in children (<18 yrs) than in adults (18-25 yrs) (36.1% vs. 18%, respectively, *p* < 0.001). Controlled for age and gender, the direct relationship between SBP, DBP, and BMI *z*-scores was found (*r* = 0.227 and *p* < 0.001 and *r* = 0.139 and *p* < 0.001, respectively).

In the whole cohort, 212 (24%) subjects were diagnosed with at least one microvascular complication. Retinopathy was diagnosed in 10.8% (*n* = 94), neuropathy in 11.5% (*n* = 97), and elevated AER in 9.4% (*n* = 83) of participants.

Glycemic control, duration of diabetes, and microvascular complications were significantly related. Hypertension was more frequent among patients with elevated AER. Prevalence of dyslipidemia was higher among patients with neuropathy. Comparison of patients according to the presence of diabetes complications is shown in Table 4.

Patients with an altered lipid profile had higher frequency of at least one microvascular complication compared to patients with normal levels of lipids (27.2 vs. 18.8%, respectively, *p* = 0.005); the same trend was found in patients with hypertension vs. patients with normal BP (25.9 vs. 23.2%, respectively, *p* < 0.001).

## 4. Discussion

Here, we present population-based study results for metabolic control, obesity, and hypertension in young people under the age of 25 with T1D treated by intensive insulin therapy, whose diabetes duration was more than 6 months.

The principal finding of our study is an unusually high frequency of dyslipidemia among pediatric and young adult patients with T1D compared to previously published data from other studies reporting dyslipidemia in 3.8% to 30.3% of subjects with T1D [7, 20]. These striking differences in the prevalence of dyslipidemia might partly be explained by different cutoff levels of lipids used in different studies. In some reports, ADA and National Cholesterol Education Program recommendations were used, defining dyslipidemia as TG level > 1.7 mmol/l, LDL cholesterol level > 3.36 mmol/l, HDL cholesterol level < 1.03 mmol/l, or total cholesterol > 5.17 mmol/l [20]. In other studies, dyslipidemia was defined as physician-diagnosed and recorded in medical documentation [7]. In our study, we used the ISPAD guidelines for the definition of optimal lipid levels [14]. Therefore, the use of different guidelines and normative data is certainly affecting the reported frequency of dyslipidemia in patients with T1D, and the comparisons between such studies are limited.

Interestingly, dyslipidemia in our study was present with similar frequency in all age groups and was directly related to diabetes duration and worse glycemic control that were independent factors influencing the occurrence of dyslipidemia in our cohort. Only after adjustment for diabetes duration and glycemic control was lipid fraction concentrations found to have a weak association with adiposity expressed in BMI *z*-scores. However, in our study, we reported slightly lower frequency of weight problems among young type 1 diabetics compared to that in recent studies from USA and Europe [7, 21]. Therefore, the overweight and obesity could not explain high rates of dyslipidemia in our cohort. On the contrary, the frequency of overweight youth among T1D patients seems to increase over time, as in the present study it was found to be higher (19.5%) than what was reported in 2013 in patients with T1D in Lithuania (13.4%) [22]. Furthermore, overweight and obese patients in our study had worse glycemic control and higher frequency of hypertension compared to normal-weight subjects, which is compatible with the results reported in other studies highlighting obesity among diabetic patients becoming a considerable health problem, affecting both adults and children [7, 21].

We found a significantly higher waist-to-height ratio in overweight and obese groups and in those with at least one lipid fraction out of the normal values. The waist-to-height ratio appeared to be a more sensitive parameter in defining risk of obesity and dyslipidemia than the waist-hip ratio, traditionally used in an adult population in the definition of central obesity. Several studies showed that the waist-to-height ratio is a better indicator than BMI and the waist-hip ratio for evaluating obesity and predicting risks for diabetes, hypertension, and CVD [11, 23]. The cutoff of 0.5 for the waist-to-height ratio was proved to be optimal in detecting abdominal obesity, metabolic syndrome, and the associated health risks [23].

We reported the mean HbA1c of 8.5 ± 2% in the whole cohort, which is markedly higher than the recommended optimal glycemic control. We found that young females have worse glycemic control than males, which is consistent with other studies' results [7, 24]. Of all age groups, patients aged 15-19 yrs have the worst glycemic control, probably because of adjustments in the endocrine system, and increased independence in diabetes care during adolescence makes achieving optimal HbA1c really difficult [25]. We observed that dyslipidemia was directly related to HbA1c levels. A positive correlation was found between the level of HbA1c and that of total cholesterol, LDL cholesterol, and Tg. These findings are in agreement with data from previous studies and provide further evidence that good glycemic control in young age would have a positive impact on lipid metabolism [26, 27].

Our analysis showed that levels of HbA1c and all lipid fraction concentrations were significantly related to the duration of diabetes. The link between T1D duration and microvascular complications and other CVD risk factors has been discussed by other authors [20, 28]. Previously published data from a study in Lithuanian T1D patients showed positive associations between the duration of diabetes and levels of total cholesterol, LDL cholesterol, and triglycerides [22]. Diabetic nephropathy was reported as the most common diabetic complication, with longer diabetes duration having a significant impact for the development of microalbuminuria [20].

Dyslipidemia in T1D has been shown to be associated with the early development of cardiac and vascular abnormalities [6]. Cholesterol is well known to be one of the key players in the process of atherosclerosis [29]. Epidemiological studies in diabetic patients have shown that increased LDL and decreased HDL cholesterol levels are associated with an increased cardiovascular risk [30]. In accordance with other studies, increased LDL cholesterol levels were the most prevalent and hypertriglyceridemia the least prevalent lipid abnormality in our cohort of young patients with T1D [31]. High levels of triglycerides were shown to be accompanied by a high rate of microangiopathic alterations [31]. Since poor glycemic control can result in increased levels of triglycerides and LDL and decreased HDL cholesterol levels, optimization of glycemic control is essential in controlling lipid levels [30].

Physical activity, smoking, alcohol consumption, and dietary habits were not included in our analysis, constituting one of the limitations of this study; however, studies show the importance of these factors for CVD risk in T1D [6]. Smoking was reported to be a significant player in the progression of atherosclerotic changes of arteries in the Pittsburgh Epidemiology of Diabetes Complications Study [32]. It is known that physical activity positively affects BP, lipid profile, and weight; therefore, it is necessary to decrease physical inactivity in T1D patients [6, 32].

Even though ADA and AHA have clinical recommendations for preventing dyslipidemia in youth with diabetes, there is still lack of clinical trial data on treatment efficacy and safety of dyslipidemia in these patients [33]. Recent data reviews suggest that dyslipidemia is one of the potentially modifiable CVD risk factors; therefore, there is a need for clinical trials to examine the safety and efficacy of lipid-lowering drugs and their impact on future health outcomes [33, 34].

In our cohort, we found a higher incidence of hypertension (29.8%), especially in children, compared to other authors [7, 23]. This high frequency of hypertensive patients might possibly be explained by unrecognized “white-coat” hypertension, whose prevalence in population-based studies was found to be up to 29.2%, and it is suggested that white-coat hypertension could be present in about one-third of subjects with high blood pressure [35]. This type of hypertension may be identified using BP monitoring at home, elucidating the frequency of real hypertension.

We found a significant correlation between SBP, DBP, and BMI *z*-scores. Our results support findings from other studies that weight management is one of the principal strategies to lower the risk of CVD in T1D patients. In the future, it is expected that metformin would also bring some benefits for obese youth with T1D [36, 37].

We report here similar frequency of microvascular complications among young T1D patients, compared to recently published data [20]. Higher frequency of hypertension was found in patients with elevated AER. Hypertension is one of the key risk factors for the development of nephropathy, and management of BP is essential in reducing the risk of kidney damage [20].

We discussed several limitations of our study. We did not perform BP monitoring at home, which would have elucidated the real prevalence of hypertensive and prehypertensive patients. Furthermore, in our cohort, we did not assess apolipoprotein B concentrations and carotid artery intima-media thickness, which are both significant predictors of CVD risk [38].

Finally, the findings of our study highlight that the management of T1D should be multifaceted and most importantly include glycemic control, weight management, and dyslipidemia treatment.

## Figures and Tables

**Table 1:** Clinical characteristics according to weight group.

	Weight group			
	Normal weight % (*N*)	Overweight % (*N*)	Obese % (*N*)	Underweight % (*N*)
All	75.8 (666)	19.5 (171)	3.6 (32)	1.1 (10)
Using insulin pumps	30.6 (204)	30.4 (52)	25 (8)	0
Optimal glycemic control	34.5 (230)	27.5 (47)	21.9 (7)	30 (3)
WtHR ≥ 0.5	11.1 (74)	50.9 (87)^∗^	84.4 (27)^∗^	10 (1)
Dyslipidemia	61.4 (408)	66.7 (114)	68.8 (22)	60 (6)
Hypertension	25.6 (168)	40.6 (69)^∗^	65.6 (21)^∗^	50 (5)
Microvascular complications				
Retinopathy	11 (72)	11.7 (20)	6.3 (2)	0
Neuropathy	9.6 (63)	17 (29)^∗^	12.5 (4)	10 (1)
Elevated AER	10.2 (66)	8.5 (14)	9.4 (3)	0

HbA1c: glycosylated hemoglobin; WtHR: waist-to-height ratio; AER: albumin excretion rate. ^∗^*p* < 0.05, compared to the normal-weight group.

**Table 2:** HbA1c levels between genders in different age groups.

Gender	Males	Females
Age group	Mean HbA1c (%)	Mean HbA1c (%)
1-4 yrs	7.3 ± 1.2	7.2 ± 0.5
5-9 yrs	7.3 ± 1.1^∗^	7.8 ± 1.3^∗^
10-14 yrs	8.2 ± 1.7^∗^	8.7 ± 2^∗^
15-19 yrs	8.6 ± 2.1^∗^	9.2 ± 2.2^∗^
≥20 yrs	8.4 ± 1.9^∗^	8.7 ± 2^∗^

HbA1c: glycosylated hemoglobin. ^∗^*p* < 0.001 comparing HbA1c between males and females for the same age group.

**Table 3:** Logistic regression: analysis of predictors on “Dyslipidemia”.

Predictor	*β*	SE *β*	Wald's *χ*^2^	*p*	OR	95% CI	
						Lower	Upper
Constant	-3.525	0.813	18.793	<0.001	0.029	NA	
HbA1c	0.207	0.042	24.632	<0.001	1.229	1.133	1.334
WtHR	5.113	1.674	9.327	0.002	166.153	6.244	4421.299

HbA1c: glycosylated hemoglobin; WtHR: waist-to-height ratio; *β*: coefficients estimated from the data; SE: standard error; OR: odds ratio; CI: confidence interval; NA: not available. Cox and Snell *R*^2^ = 0.046; Nagelkerke *R*^2^ = 0.063.

**Table 4:** Clinical characteristics according to presence of diabetic microvascular complications.

	Retinopathy		Neuropathy		AER	
	Absent	Present	Absent	Present	Normal	Elevated
Duration of DM (yrs)	6 ± 4.2	12.8 ± 4.4	6.2 ± 4.5	11 ± 4.7	6.4 ± 4.6	9.3 ± 5.7
	*p* < 0.001		*p* < 0.001		*p* < 0.001	

HbA1c (%)	8.3 ± 1.8	10 ± 2.3	8.4 ± 1.9	9.3 ± 2.3	8.4 ± 1.9	9.5 ± 2.4
	*p* < 0.001		*p* < 0.001		*p* < 0.001	

% of participants with dyslipidemia	61.9	70.2	61.4	74.2	62.1	69.9
	*p* = 0.117		*p* = 0.014		*p* = 0.164	

% of participants with hypertension	31.2	23.7	30.4	30.2	29.2	41.5
	*p* = 0.083		*p* = 0.9		*p* = 0.031	

DM: diabetes mellitus; HbA1c: glycosylated hemoglobin; AER: albumin excretion rate.

## Data Availability

The data used to support the findings of this study are available from the corresponding author upon request.
